# Mechanisms that initiate ventricular tachycardia in the infarcted human heart

**DOI:** 10.1016/j.hrthm.2009.09.025

**Published:** 2010-01

**Authors:** Oliver R. Segal, Anthony W.C. Chow, Nicholas S. Peters, D. Wyn Davies

**Affiliations:** St. Mary's Hospital and Imperial College, London, United Kingdom

**Keywords:** Arrhythmia, Electrophysiology, Infarction, Reentry, Tachycardia, Ventricles, DP, diastolic pathway, ECG, electrocardiogram, ICD, implantable cardioverter defibrillator, LV, left ventricle, MI, myocardial infarction, VT, ventricular tachycardia

## Abstract

**Background:**

Precise mechanisms that initiate ventricular tachycardia (VT) in the intact infarcted human heart have not been defined.

**Objective:**

The purpose of this study was to investigate the mechanisms that underlie human postinfarct VT initiation.

**Methods:**

Noncontact mapping of the left ventricle was performed in 9 patients (age 67.1 ± 7.8 years, ejection fraction 34.4% ± 5%) with previous myocardial infarction and sustained monomorphic VT.

**Results:**

Circuits in which ≥30% of the diastolic pathway (DP) could be defined were identified in 12 VTs (cycle length 357 ± 60 ms). Eighteen VT episodes were initiated with pacing, and one occurred spontaneously. Ten complete and two partial circuits were mapped (89% ± 25% of the DP). In all complete circuits, pacing led to the development of unidirectional conduction block at the location of the subsequent VT exit site and the formation of functional block creating a border(s) for subsequent DP. Wavefront velocity in the DP region slowed from 1.22 ± 0.2 m/s during sinus rhythm to 0.59 ± 0.14 m/s during VT (*P* <.005). In 11 initiation episodes, lines of functional block and areas of slow conduction developed progressively over one to six reentrant cycles before a stable DP was established and sustained monomorphic VT ensued. The formation of unidirectional or functional lines of block was not identified during identical pacing protocols that failed to initiate VT (n = 14).

**Conclusion:**

Initiation of sustained monomorphic VT requires the development of unidirectional block and formation of lines of functional block creating borders for a DP in areas of slow conduction. A transitional stage often exists during the initiation process before a stable VT circuit is established.

## Introduction

Sustained monomorphic ventricular tachycardia (VT) affects up to 5% of patients with remote myocardial infarction,^1^ of which the underlying mechanism in the majority of cases is reentry.^2–4^ Programmed ventricular stimulation is widely used for the initiation of sustained monomorphic VT and traditionally was considered a useful predictor of future arrhythmic events and mortality following myocardial infarction.^5^ Premature extrastimuli delivered to the right ventricle can induce VT in up to 70% of patients with documented spontaneous VT,^6^ increasing to 90% with left ventricular (LV) stimulation.^7^ Experimental and animal models have implicated a number of different factors necessary for the initiation of stable monomorphic VT, including formation of lines of functional block,^3,8^ development of a region of slow conduction,^4^ formation of unidirectional block,^9^ anisotropy,^10^ and dispersion of tissue refractoriness leading to reentry.^11^ The precise role of these factors in the initiation of spontaneous or pacing-induced VT in the intact human heart in patients with remote myocardial infarction remains to be fully determined. Greater insight into the mechanisms that underlie VT initiation may improve our ability to identify patients likely to develop VT in the future, permit more selective prescription of implantable cardioverter-defibrillator therapy, and help devise interventions to treat or prevent the spontaneous occurrence of VT.

## Methods

Consecutive patients with a history of remote myocardial infarction (>6 months) and spontaneous episodes of sustained monomorphic VT despite treatment with antiarrhythmic drugs underwent noncontact mapping–guided ablation. The study was approved by the local ethics committee, and patients provided written informed consent.

### Mapping procedure

High-resolution activation maps of the entire LV endocardium were created using noncontact mapping (EnSite 3000, St. Jude, St. Paul, MN, USA),^12^ with the multielectrode array deployed retrogradely via the aorta. Intracardiac contact electrograms and surface 12-lead ECG were recorded on a conventional electrophysiology system ((Bard EP Lab System, Lowell, MA, USA) and bipolar electrograms filtered at 30 to 500 Hz. A quadripolar catheter deployed in the right ventricle was used to induce VT using burst pacing, double ventricular extrastimuli, or up to three extrastimuli following eight-beat drive trains (S1 600 and 400 ms). Intracardiac 4-mm nonirrigated mapping/ablation catheters (Cordis, Biosense Webster, Diamond Bar, CA, USA) were deployed retrogradely and via transseptal puncture. Patients were given heparin, with the activated clotting time maintained between 300 and 400 seconds.

Isopotential and isochronal maps and electrograms were analyzed at multiple filter settings to ensure the consistency of patterns of activation and electrogram fidelity. Distance measurements obtained using the noncontact system were calculated to account for surface curvature, as described previously.^13^ VT circuits in which >30% of the diastolic pathway could be identified were included for analysis.

### Definitions

*Regions of scar* were defined as areas of endocardium with absent or very-low-amplitude reconstructed electrograms during sinus rhythm, pacing, *and* VT. Scar was confirmed in these regions using bipolar contact catheters demonstrating electrogram amplitudes <0.5 mV. Although some investigators have defined normal endocardium as areas with bipolar electrograms >1.5 mV,^14^ other investigators have chosen <0.5 mV to define true scar, with infarct border zone tissue lying between these two values.^15,16^ Using the former definition would have reduced the infarct border zone size, the principal area of interest for the study. We previously used a similar definition.^17^

*VT exit sites* were defined as points of rapidly expanding systolic activation on the isopotential map synchronous with or just prior to QRS onset.

*Lines of functional block* were defined as lines that divided activation between adjacent endocardial areas by >50 ms, were not fixed (seen with pacing or tachycardia only), and varied with different rates of ventricular activation. When present, they produced dissociated activation in adjacent regions and electrograms with double potentials. They were confirmed by identification with pacing from different sites.

*Diastolic pathways* (DPs) were defined as regions of the LV that activated between QRS complexes during VT and were protected from systolic activation by lines of block or scar.

*Transitional beats* were defined as cycles of ventricular activation occurring immediately prior to the establishment of sustained monomorphic VT with different surface QRS morphology and endocardial activation patterns from monomorphic VT.

### Statistical analysis

Continuous data are presented as mean and standard deviation and were compared using the Wilcoxon paired test. Data analysis was performed using SSPS 10.0 statistical software (SPSS, Inc., Chicago, IL, USA). *P* <.05 was considered significant.

## Results

In 9 patients (7 men and 2 women; age 67.1 ± 7.8 years) with myocardial infarction (4 anterior, 3 inferior, 1 posterior, 1 anterior and inferior) and impaired LV function (ejection fraction 34.4% ± 5%), complete episodes of VT initiation were recorded and at least 30% of the DP, including entry and exit sites, identified (Table 1). Only in two VT circuits could <100% of the DP be identified. All patients were treated with amiodarone, and four had been treated with mexiletine (Table 1).

### Episodes of VT initiation

Nineteen episodes of VT initiation (18 induced by programmed stimulation, 1 occurred spontaneously) with 12 different VT morphologies (cycle length 357 ± 60 ms) were recorded. Four of the VTs were initiated during continuous pacing (420 ± 98 ms, range 300–500 ms), 2 were initiated after delivery of closely coupled ventricular extrastimuli during sinus rhythm (280 ms/280 ms and 280 ms/260 ms), and the remaining 12 were induced with one to three premature ventricular extrastimuli (range 240–360 ms) following a drive train of eight paced beats (S1 400–600 ms). Complete circuits (100% of DP) were mapped in 10 VTs, and partial DPs (>30%) were identified in 2 VTs (overall 89% ± 25% of all DPs, range 31%–100%). Completely mapped DPs were 5.2 ± 2.1 cm long.

**Figure 1:**
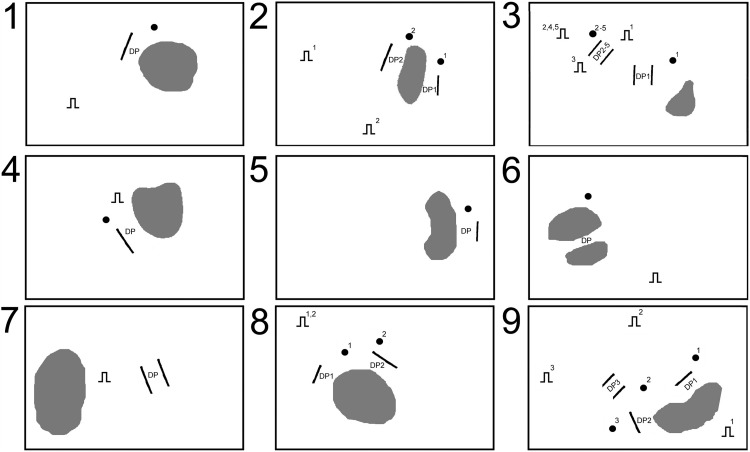
Schematic representation of left ventricular endocardium in all nine patients showing infarct scar (*gray*), pacing site location (*black pacing symbols*), diastolic pathway (DP; *paired black lines*), and transitional reentry zones. *Numbered black dots* indicate sites of the corresponding evolving transitional beat diastolic pathway. Patients 2, 3, and 9 have ≥2 pacing symbols representing the position of pacing for separate ventricular tachycardia episodes. See text for discussion.

Figure 1 shows a schematic representation of the LV endocardium in all nine patients. Infarct scar is shown in gray. Black pacing symbols show pacing site locations. Patients 2, 3, and 9 have ≥2 pacing symbols representing the position of pacing for separate VT episodes. Paired black lines represent DPs. Numbered black dots indicate sites of the corresponding evolving transitional beat DP.

### DP formation

Borders of the DPs of monomorphic VT were formed by a mixture of anatomic (infarct scar) and functional block in 7 VTs, purely by lines of functional block in 4 VTs, and entirely bordered by two areas of fixed block in 1 VT.

In all cases of VT initiation, unidirectional block formed at the same location as the subsequent monomorphic VT exit site, thereby protecting an area of myocardium that formed the DP. The activation wavefront was forced to propagate around this line of block, enter the protected area at the same location as the subsequent entry site of the monomorphic VT DP, and then propagate through this region toward the exit site region. Upon reaching the distal end of this protected region, the wavefront of activation was able to continue because either conduction block was unidirectional or tissue was no longer refractory. This led to the development of stable reentry.

**Figure 2:**
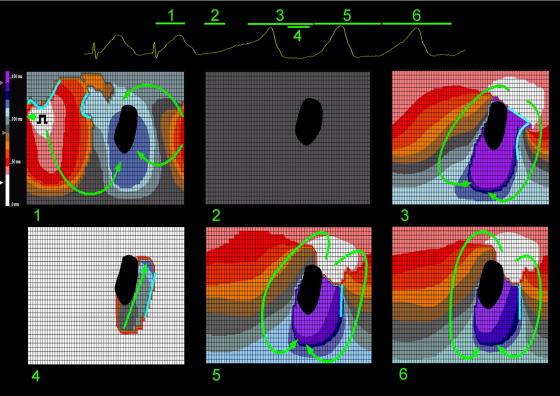
Sequence of noncontact left ventricular isochronal maps demonstrating an episode of pacing-induced ventricular tachycardia initiation from patient 2. *Flat maps* represent the entire left ventricular endocardium cut along one border and laid open. *Black region* represents scar. *Light blue lines* represent lines of functional block. *Arrows* show direction of propagation of activation. Change of isochrone color represents 10-ms intervals in activation, progressing from *white* to *purple* (illustrated on the scale adjacent to panel 1). The corresponding surface ECG is shown at the **top** with *numbers* pertaining to map panels below and *lines* representing the point in time and duration of each map. In this and all subsequent figures, the base of the left ventricle is at the top of each panel and the apex at the bottom. See text for discussion.

An example of this phenomenon is shown in the sequence of isochronal maps in Figure 2 recorded from patient 2. The flat maps represent the entire LV endocardium cut along one border and laid open. The black region represents scar, light blue lines represent lines of functional block, and arrows show direction of propagation of activation. Change of isochrone color represents 10-ms intervals in activation, progressing from white to purple (illustrated on the scale adjacent to panel 1). The corresponding surface ECG is shown at the top with numbers pertaining to map panels below and lines representing the point in time and duration of each map. In this and all subsequent figures, the base of the LV is at the top of each panel and the apex at the bottom.

The first isochronal map (panel 1) represents the last paced beat from a 300-ms drive train. No lines of block are present around the infarct border zone, although one is present adjacent to the pacing site. Following a period of electrical silence (panel 2), endocardial activation originates in the posterobasal position (white area, panel 3) adjacent to scar. Although this period of apparent electrical silence could represent very slow conduction through scar (not seen) or through epicardial or intramural myocardium (not detectable by the noncontact system), conduction time for this period is approximately 500 ms, but the subsequent VT cycle length is only 360 ms. A line of block prevents direct penetration of the infarct border zone, and activation travels in a figure-of-eight pattern around both scar and a functional line of block parallel to scar border (panel 3). Conduction through the entry point and the rest of the protected region is slow, demonstrated by the tightly packed isochrones (panels 3 and 4). Recovery of excitability occurs at the distal end of the protected region, enabling sustained reentry (panels 5 and 6).

Initiation of the single spontaneous episode of VT in the study (in patient 5) shared some similar characteristics. A single premature ventricular ectopic beat originating near the VT exit site caused arcs of functional block to form a protected region, which subsequently acted as the DP.

### Transitional beats

A total of 37 transitional beats (1.9 ± 1.5 beats per VT episode, range 0–6 beats) were observed occurring immediately after programmed stimulation in 16 of 19 successful VT initiations prior to the establishment of sustained monomorphic VT. In the remainder, sustained monomorphic VT occurred immediately after pacing.

Sixteen transitional beats were identified at discrete sites immediately after programmed stimulation, 9 adjacent to the pacing site and 7 at sites distant from the pacing site. The remaining 18 transitional beats occurred due to reentry through the region of the subsequent DP. These regions were bordered by lines of functional block that changed in size and position from cycle to cycle. Duration of transitional reentry was one to two cycles before stable lines of block were established, protecting the DP. All transitional DPs developed adjacent to sites of the subsequent stable DP of the monomorphic VT circuit.

**Figure 3:**
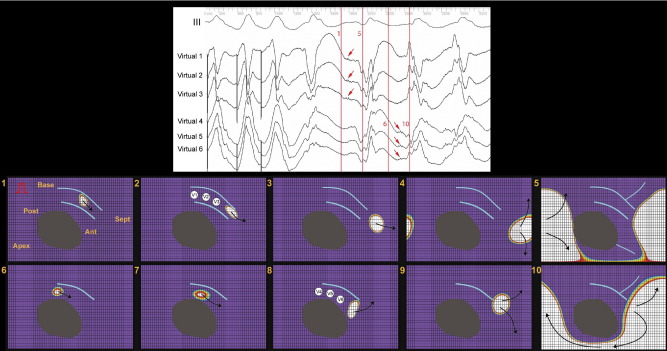
Sequence of noncontact left ventricular isopotential maps from patient 8 demonstrating a transitional beat prior to monomorphic ventricular tachycardia. Flat isopotential maps represent the entire left ventricle cut along one border and laid open. *Gray areas* are infarct scar. *Red rectangular symbol* is the pacing site. *Purple* represents resting endocardial potential that changes through a spectrum of colors on activation, with *white* representing maximal change of negative potential. *Arrows* show direction of activation. *Light blue lines* represent lines of functional block. Panel at the **top** shows surface ECG lead III with reconstructed electrograms beneath (Virtual 1–6). *White circles* in panels 2 and 8 indicate sites where reconstructed electrograms were recorded. A ventricular premature beat occurs after S3, then a transitional beat and then sustained monomorphic ventricular tachycardia. *Vertical red lines* are lettered and correspond with the panels below. See text for discussion.

An example of a transitional beat with reentry through the evolving DP is shown in Figure 3 (patient 8). A series of isopotential maps is displayed rather than isochrones because the version of the software used for this patient only permitted automated electrogram timing for isochronal maps leading to inaccuracy. The flat isopotential maps represent the entire LV cut along one border and laid open. Gray areas are infarct scar, and the red rectangular symbol is the pacing site. The purple color represents resting endocardial potential that changes through a spectrum of colors on activation, with white representing maximal change of negative potential. Arrows show direction of activation, and light blue lines represent lines of functional block. The panel at the top shows surface ECG lead III with reconstructed electrograms beneath (Virtual 1–6). The white circles in panels 2 and 8 indicate sites where reconstructed electrograms were recorded. A ventricular premature beat occurs after S3, then a transitional beat and then sustained monomorphic VT. Vertical red lines are lettered and correspond with the panels below.

Panels 1 through 5 demonstrate diastolic activity and the start of systole during the transitional beat. Two lines of functional block have formed a protected channel in the anterobasal peri-infarct region. Panels 1 and 2 show activation proceeding through this channel before exiting through the midseptum and across the posterior wall (panels 3–5). Panel 5 shows a functional line of block extending from scar in the anteroapical region. Diastolic electrograms are present on Virtual 1–3 (red arrows).

Panels 6 through 8 demonstrate the same part of the cardiac cycle for the first beat of sustained VT. In contrast with the transitional beat, a single line of functional block in the anterobasal region protects a channel of myocardium between it and infarct scar. Diastolic activity is shown in panels 6 through 8, the exit site in 9, and systolic activation in panel 10. Again, diastolic electrograms are present, shown on Virtual 4–6 (red arrows). The functional line of block present during systole in panel 5 is not present in panel 10, thus accounting for the different systolic activation pattern and surface ECG appearance. No further evolution of the DP borders occurred, and VT was sustained and monomorphic.

### Wavefront velocity

Significant slowing of wavefront velocity was found within the DP during reentry. Wavefront velocities measured at the same region during sinus rhythm were 1.2 ± 0.2 m/s, which slowed to 0.59 ± 0.14 m/s during VT (*P* <.005).

### Failed VT inductions

Thirty-four episodes in which similar or identical stimulation sequences failed to induce VT were recorded and analyzed. Eighteen episodes ended in failure because the line(s) of functional block extended directly from scar, thereby preventing the formation of a protected region with slow conduction properties (see below). In 1 episode, conduction block failed to form. In 11 episodes, lines of functional block developed to form transitional DPs but rapidly destabilized, preventing reentry. In the remaining 4 episodes, VT failed to initiate despite formation of a protected region of myocardium bordered by lines of block due to bidirectional rather than unidirectional block occurring at the VT exit site. All sites at which pacing failed to initiate VT were located within the LV, and pacing from the same site successfully initiated VT using different pacing intervals.

**Figure 4:**
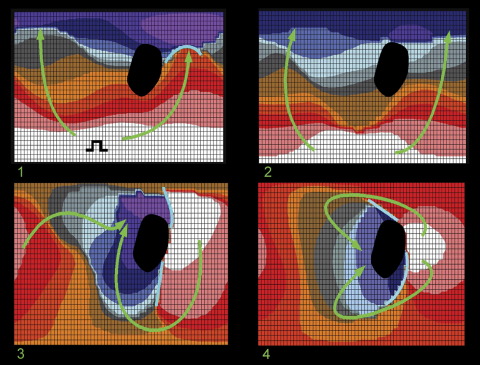
Sequence of noncontact left ventricular isochronal maps showing failure of ventricular tachycardia initiation due to formation of only a single line of functional block in patient 7. See text for discussion. Change of isochrones color represents 10-ms intervals in activation, progressing from white to purple.

An example of a failed VT induction due to formation of lines of block extending directly from scar is shown in Figure 4 (patient 7). The first panel shows the last extrastimulus (S3 290 ms following a drive train) originating at the apical LV. Infarct scar is shown in the posterobasal region, and a small line of functional block is associated with the lateral part of the infarct border zone. The second panel shows the first postpacing beat originating from the same area as the pacing site, again with activation spreading basally, but the line of functional block has disappeared. The next point of endocardial activation then occurs at the lateral border of the infarct scar (panel 3). Of note, two lines of block form at the lateral border extending from the scar basally and apically. The final postpacing beat (panel 4) originates from the same site as in panel 3, resulting in some extension of the lines of block, but a protective channel has not formed; therefore, reentry cannot develop (compare Figure 2, panel 5, and Figure 4, panels 3 and 4).

### Ablation results

All 12 VT morphologies induced in the present study were targeted for ablation. Radiofrequency energy was delivered to sites within the DP identified on noncontact maps and confirmed by entrainment mapping. Of these morphologies, 8 occurred spontaneously prior to the ablation procedure. During follow-up of 33.2 ± 18.5 months, 4 patients died, 2 patients had a recurrence of a targeted VT and required repeat ablation, 1 patient developed new VT, and 1 patient experienced VF and was successfully resuscitated.^18^

## Discussion

In this first detailed description of the mechanisms responsible for infarct-related VT initiation in the intact human heart using global endocardial three-dimensional mapping, results demonstrate that VT circuits are dependent on reentry via a protected DP, typically bounded by a combination of fixed and functional block. Purely functional and purely anatomically determined reentrant circuits (reentry through scar) both were identified, although the latter was observed only once.

In the majority of cases, development of functional block is essential for initiation of reentrant VT, by protecting a region of myocardium that subsequently forms the DP. Conversely, if lines of functional block do not develop or a protected channel is not formed, VT fails to initiate. Destabilization of functional lines of block with failure to form a protected DP channel also leads to failure of VT initiation.

### Comparison with previous studies

Detailed study of VT initiation previously has been possible only using *in vitro* or *in vivo* models^19,20^ or during surgical mapping studies.^3,4^ In the present study of the intact heart, burst pacing or ventricular extrastimuli resulted in the formation of functional block at the region that became the subsequent DP exit site, and. in the majority of cases, a line of bidirectional block formed a border of the subsequent DP. In combination, these lines of block created a protected zone of slow conduction allowing recovery of myocardial excitability essential for reentry.

Mean VT cycle length in the present study was 357 ± 60 ms compared with 439 ± 76 ms in a study by Hsia et al,^21^ 365 ± 77 ms in a study by Arenal et al,^22^ and 396 ± 124 ms in a study by Bogun et al.^23^ It is possible that as VT cycle length in the present study is shorter, these circuits may contain a greater proportion of functional rather than anatomic block. Alternatively, it could reflect a shorter circuit wavelength. Against, this would be the relatively longer DP length: 5.2 ± 2.1 cm in the present study versus 2.3 ± 1.1 cm reported by Arenal et al^22^ and 3.1 ± 2.1 cm reported by Hsia et al.^21^ This latter disparity may reflect different methods of measuring the DP. In the present study, entrance and exit sites were included.

### Importance of functional block in human VT

Traditionally, fixed anatomic barriers (e.g., scar) have been considered critical for reentrant VT circuits.^24^ However, observations from this and other studies suggest that functional block is necessary, at least in part, for the formation of the majority of human^3,4,13,25^ and canine VT DPs.^26^ *In vivo* evidence indicates that formation of functional block leading to reentry is associated with large dispersion in refractory periods over short anatomic distances.^9,27^

In the present study, formation of unidirectional block was a prerequisite for all VT initiations, and, in the majority, functional block formed at least one border of the DP. Functional block did not always develop despite short coupling intervals, suggesting that formation of functional block is dependent on direction of activation following stimulation. Furthermore, because VT could be initiated from the same site at which different coupling intervals had failed, changes in cellular electrophysiology rather than the site of stimulation *per se* appears to be the critical determinant of success or failure in those instances. A complete explanation for these phenomena is beyond the scope of this study but is likely to be attributable to spatial differences in refractoriness and anisotropic conduction.^28^

### Functional block and transitional beats

Monomorphic VT typically did not start instantaneously but usually evolved over several cycles, with intervening transitional beats. The present study showed that these beats originate from three sources: (1) discrete endocardial activation from the site of pacing, (2) remote from both pacing site and DP, and (3) as a result of reentry through an embryonic DP.

Monophasic action potential duration shortens at pacing sites compared with nonpacing sites.^29^ This would allow rapid local recovery of excitability and impulse formation due to intramural reentry. Unique to this study, transitional beats originating remote from the pacing site were seen, which subsequently became the exit site of the VT circuit. Despite a different QRS appearance, endocardial activation often closely resembled that of the ensuing monomorphic VT, suggesting that change to QRS morphology may result from changes in intramural propagation.

An important caveat to these findings is that the noncontact mapping system detects electrical activity only up to a limited depth from the endocardium. Intramural or subendocardial activation preceding stable reentry cannot be defined, and deeper parts of reentrant circuits cannot be detected by the noncontact system. It is feasible that significant parts of the DP are located within scar rather than at scar border.

Although programmed stimulation is an artificial technique for inducing arrhythmia, evidence suggests that similar mechanisms occur during spontaneous VT. Holter studies and ICD data of episodes of spontaneous VT have demonstrated that stable VT is often preceded by one to five premature beats with similar but not identical QRS morphology.^19^ Based on the data from the present study, these may represent ectopic or transitional reentrant beats.

### Implications for VT ablation

We previously showed that focal ablation to treat infarct-related VT is highly successful, but the majority of patients develop new VT or VF within 3 years.^18^ Mechanistic observations of VT initiation in the present study demonstrate that areas at which lines of functional block form are critical to VT formation. Ablation strategies that target such areas may prevent reentry and VT occurrence. This is consistent with the excellent results of studies of substrate ablation that predominantly targets infarct border zone areas.^30^ Whether such a strategy would have prevented both recurrences of a targeted VT in the present study or the development of new VT or VF is not known.

### Study limitations

The number of VT episodes in the present study was relatively small. Different mechanisms of VT initiation not seen in the studied cohort may exist, and the actual proportion of functional and anatomically based VT circuits may be different. All but one initiation was induced by programmed stimulation. Therefore, findings from the present study may not be applicable to spontaneous clinical VT initiation. Changes in tissue refractoriness cannot be determined using this mapping system, and alterations with premature stimulation are theoretical assumptions. Endocardial distances were measured as a series of straight lines. A small degree of error would have occurred due to the curved endocardial geometry, so to limit this, only short straight lines (mean 6 ± 2 mm) were measured. The difference in DP conduction during VT and sinus rhythm would have been influenced by direction of activation and nonuniformity of anisotropy. Only VTs in which >30% of the DP was mappable were included for analysis, although only two VT circuits had <100% mapped. This cutoff is arbitrary; complete DP activation was not seen for these two circuits. Interpretation of virtual electrograms using the noncontact system requires a considerable degree of experience with the system, especially when trying to discern real electrograms from background noise artifact or artifact from repolarization. We have one of the longest experiences in using this mapping system after its validation for mapping VT in 1999.

## Figures and Tables

**Table 1:** Patient characteristics and mode of VT induction

Patient no.	Age/sex	Ischemic heart disease	Drugs	Ejection fraction (%)	VT cycle length (ms)	Percent DP mapped	Mode of induction (S1) + S2 + S3 (ms)	No. of transitional beats	Discrete transitional beats at pacing site	Transitional reentrant beats
1	60/M	Yes	Am	35	335	100	(400) + 290	1	1	1
2	72/F	Yes	Am+Mex	30	360	100	(300)	1	1	0
					285	100	(400) + 260 + 260	6	1	2
3	55/M	Yes	Am	30	380	100	(600) + 260 + 260	2	0	2
					310	100	(550) + 240 + 260	2	0	2
							(550) + 240 + 260	2	0	2
							(550) + 240 + 260	2	0	2
							(600) + 260 + 260	2	0	2
4	69/M	Yes	Am+Mex	35	340	100	(400) + 360 + 360	1	0	1
5	61/M	Yes	Am+Mex	35	430	100	Spontaneous	1	0	0
6	63/M	Yes	Am+Bi	40	380	40	280 + 260	1	1	0
							280 + 280	0	0	0
7	75/M	Yes	Am	35	480	31	(600) + 300 + 290	0	0	0
8	70/M	Yes	Am+Mex	30	310	100	(500) + 300 + 260	4	3	1
					475	100	(500) + 300 + 280	3	1	2
9	79/F	Yes	Am	35	320	100	(380)	0	0	0
							(500)	2	1	0
							(500)	4	1	1
							(400) + 220 + 200	3	1	0
								37	11	18
								1.9 ± 1.5	0.58 ± 0.77	0.95 ± 0.91

Brackets around numbers indicate drive trains (S1) followed by premature extras S2 and S3. Transitional beats are polymorphic beats on ECG occurring after pacing but before stable monomorphic ventricular tachycardia (VT) is established. Am = amiodarone; Bi = Bisoprolol; DP = diastolic pathway; Mex = mexiletine.
